# Unveiling the potential of *Pseudococcomyxa simplex*: a stepwise extraction for cosmetic applications

**DOI:** 10.1007/s00253-024-13229-9

**Published:** 2024-06-24

**Authors:** Paola Imbimbo, Enrica Giustino, Alfonso Ferrara, Gerardo Alvarez-Rivera, Hassan Annaz, Elena Ibanez, Maria Chiara Di Meo, Armando Zarrelli, Daria Maria Monti

**Affiliations:** 1 Department of Chemical Sciences, University of Naples Federico II, Via Cinthia 4, 80126 Naples, Italy; 2 Laboratory of Foodomics, Institute of Food Science Research, CIAL, CSIC, Nicolás Cabrera 9, 28049 Madrid, Spain; 3 College of Agriculture and Environmental Science, AgroBioSciences Program, University Mohammed VI Polytechnic, Ben Guerir, Morocco; 4 Department of Sciences and Technologies (DST), University of Sannio, 82100 Benevento, BN Italy

**Keywords:** *Pseudococcomyxa simplex*, Carotenoids, Fatty acids, Biorefinery, Cosmetics

## Abstract

**Abstract:**

Microalgae are gaining attention as they are considered green fabrics able to synthesize many bioactive metabolites, with unique biological activities. However, their use at an industrial scale is still a challenge because of the high costs related to upstream and downstream processes. Here, a biorefinery approach was proposed, starting from the biomass of the green microalga *Pseudococcomyxa simplex* for the extraction of two classes of molecules with a potential use in the cosmetic industry. Carotenoids were extracted first by an ultrasound-assisted extraction, and then, from the residual biomass, lipids were obtained by a conventional extraction. The chemical characterization of the ethanol extract indicated lutein, a biosynthetic derivative of α-carotene, as the most abundant carotenoid. The extract was found to be fully biocompatible on a cell-based model, active as antioxidant and with an in vitro anti-aging property. In particular, the lutein-enriched fraction was able to activate Nrf2 pathway, which plays a key role also in aging process. Finally, lipids were isolated from the residual biomass and the isolated fatty acids fraction was composed by palmitic and stearic acids. These molecules, fully biocompatible, can find application as emulsifiers and softener agents in cosmetic formulations. Thus, an untapped microalgal species can represent a sustainable source for cosmeceutical formulations.

**Key points:**

• *Pseudococcomyxa simplex has been explored in a cascade approach.*

• *Lutein is the main extracted carotenoid and has antioxidant and anti-aging activity.*

• *Fatty acids are mainly composed of palmitic and stearic acids.*

**Graphical abstract:**

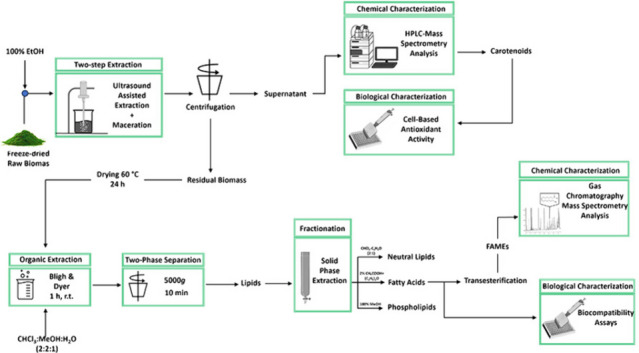

**Supplementary Information:**

The online version contains supplementary material available at 10.1007/s00253-024-13229-9.

## Introduction

Cosmetic is one of the most remunerative industrial sectors in the world, constantly growing since 2004. It has been estimated that, in 2028, the revenues of the global cosmetic market will be nearly 129 billion USD. In particular, the beauty market is dominated by skincare, which is expected to reach 221.38 billion UDS by 2030 with a CAGR of 4.79% (https://straitsresearch.com/report/facial-skincare-products-market, accessed on 14 March 2024). Nowadays, consumers prefer to use natural cosmetics that are produced from natural sources that can be considered safe for humans and environment. This trend inversion is due not only to the growing environmental consciousness of the consumers, but also to their awareness of health issues that can occur by using synthetic compounds, such as hyperactivity, allergic reactions, or other side effects (Tang et al. 2020). Thus, different cosmetic companies are using biotechnology to ensure quality, efficacy, and safety of natural products (Bouzroud et al. 2023). Among natural sources, microalgae have gained attention as they are considered green fabrics able to synthesize a plethora of bioactive metabolites, endowed with unique biological activities (Liberti et al. 2023). Moreover, they are perceived as vegan, natural, and healthy by consumers. To date, only a few strains have been exploited for cosmetic purposes, such as *Spirulina*, *Dunaliella*, *Chlorella*, and *Haematococcus* (Yarkent et al. 2020). Despite the considerable potential of microalgae, some issues still limit their full industrialization, such as high upstream and downstream costs (Imbimbo et al. 2020). To make the use of microalgae sustainable and feasible, the biorefinery approach has been proposed as the technology is able to implement the circular economy and lowers the overall process costs (Igbokwe et al. 2022).

Carotenoids are pigments which can be used in different fields, such as the food and pharmaceutical industry, and recently, due to the potent antioxidant activity, they have attracted interest to be used as active ingredients in cosmetic formulations. Currently, the market is dominated by astaxanthin, β-carotene, zeaxanthin, lutein, and lycopene (Sathasivam and Ki 2018),

Lipids, and more in general oils, are one of the major components in cosmetic cream formulations (Franco et al. 2022). Based on their nature, they can be used for different purposes, such as softener agents, emulsifiers, detergents, and skin lighteners (De Luca et al. 2021). Fatty acids are used in cosmetics as emollients to improve the skin hydration, and as emulsifiers, since they can act as thickening agents. It has been reported that the ideal length of the carbon chain for fatty acids-based emulsion is 16–18, which correspond to palmitic and stearic acid (Cochran and Anthonavage 2015). Fatty acids are also physiological skin components that ensure the maintenance of skin barrier functions (Knox and O’Boyle 2021). In this paper, we proposed a biorefinery approach starting from the biomass of the green microalga *Pseudococcomyxa simplex* for the extraction of two classes of molecules with a potential use in cosmetic industry. Carotenoids were extracted as the first class of molecules by an ultrasound-assisted extraction coupled to maceration, and then, from the residual biomass, lipids were obtained by a conventional extraction. Finally, a chemical and biological characterization of both classes of molecules has been carried out to investigate a possible use of these molecules in cosmetic field.

## Materials and methods

### Reagents

All the reagents, unless differently specified, were purchased from Sigma-Aldrich (Milan, Italy).

### Microalgae strain and cultivation

*Pseudococcomyxa simplex* (ACUF 127) was provided by the Algal Collection of the University Federico II (ACUF, www.acuf.net). The cultivation was carried out in bubble column photobioreactors in Bold Basal Medium (BBM) at 24 ± 2 °C with a constant light intensity of 100 PARs [(μmol_photons_/m^2^)/s]. The culture was mixed by bubbling air through a sintered glass tube placed at the bottom of each reactor. Algal growth was monitored by measuring the absorbance at 730 nm.

The dry weight determination was carried out via conversion between the Optical Density (O.D.) and the biomass dry weight at the end of the exponential growth phase. The conversion factor was 1 O.D. corresponded to 0.22 mg dry weight. The biomass concentration achieved at the end of the exponential growth phase was 0.7 g_D.W._/L.

### Pigments extraction and characterization

Pigments were extracted using ethanol as solvent, as previously reported (Imbimbo et al. 2023). Briefly, 200 mg of dry weight (D.W.) of alga were suspended in 4 mL of pure ethanol and disrupted by ultrasonication (40% amplitude, 4 min on ice, Bandelin SONOPULS HD 3200, tip MS73). The volume was adjusted to 20 mL and the mixture was shaken for 24 h at 250 rpm at 4 °C in the dark. Pigments were recovered in the supernatants by centrifugation at 5000 *g* for 10 min, and then, ethanol was removed under N_2_ stream. The extraction yield was determined gravimetrically.

Carotenoids and pigment identification was performed by HPLC–DAD-APCI-QTOF-MS/MS. The analysis of the extracts was carried out in an Agilent 1290 UHPLC system (ultrahigh performance liquid chromatography) equipped with a diode-array detector (DAD), coupled to an Agilent 6540 quadrupole-time-of-flight mass spectrometer (q-TOF MS) equipped with an atmospheric pressure chemical ionization (APCI) source. A Thermo Fisher Scientific Accucore C30 column (2.6 μm, 4.6 × 50 mm) was used at 30 °C. Separation was achieved using a 12-min gradient program from 100% mobile phase A (90% methanol, 7% MTBE, 3% water) and 0% mobile phase B (90% MTBE, 10% methanol) to 0% mobile phase A and 100% mobile phase B, and kept constant for 1.5 min before returning to the initial conditions within 1.5 min. The total run time was 15 min at a flow rate of 0.8 mL/min. The mass spectrometer was operated in positive ionization mode (APCI +), with gas temperature at 300 °C; drying gas at 8 L/min; vaporizer temperature at 350 °C; nebulizer pressure at 40 Psi; capillary voltage at 3500 V; corona + : 4 μA; fragmentor voltage at 110 V; and skimmer voltage at 45 V. The MS and auto MS/MS modes were set to acquire *m/z* values ranging between 25 and 1500, at a scan rate of 10 spectra per second. Auto MS/MS mode was operated at two collision-induced dissociation energies: 20 and 40 eV and selecting 4 precursor ions per cycle at a threshold of 200 counts.

### Lipids extraction and characterization

Lipids were recovered by using the original Bligh and Dyer protocol (Bligh and Dyer 1959). Extractions were carried out on 300 mg of both freeze-dried biomass and residual freeze-dried biomass (i.e., after pigment extraction) using chloroform, methanol, and water in a ratio 2:2:1 (v/v/v) for 1 h at room temperature. At the end of the extraction, the hydrophobic phase was recovered by centrifugation and the extract was dried under N_2_ stream. The lipid extract was then fractionated in three different lipid classes (i.e., neutral lipids, fatty acids, and phospholipids) by performing a solid-phase extraction (SPE) as previously described (Imbimbo et al. 2019). The recovered fatty acids were first derivatized by transforming them into the corresponding methyl esters and then identified and quantified by GC − MS analysis (Crescenzo et al. 2015).

### Biological characterization

#### In vitro anti-aging assays

The in vitro anti-aging activity was evaluated by anti-tyrosinase and anti-elastase assays, as described by Mahdi et al. (Mahdi et al. 2024). Kojic acid was used as a commercial inhibitor. Values are reported as inhibitory concentration (IC_50_), which represents the concentration of molecule able to inhibit 50% of the enzyme activity and expressed as milligram *per* milliliter (mg/mL).

#### Cell culture and biocompatibility of ethanol extract

Immortalized human keratinocytes (HaCaT) were from Innoprot (Biscay, Spain) and immortalized murine fibroblasts (BALB/c-3T3) were from ATCC (VA, USA). Cells were cultured in Dulbecco’s modified Eagle’s medium (DMEM), supplemented with 10% fetal bovine serum (HyClone), 2 mM l-glutamine, and antibiotics, under a 5% CO_2_ humidified atmosphere at 37 °C. The biocompatibility of either ethanol extract or FAs was tested. HaCaT cells were seeded in 96-well plate at a density of 2.5 × 10^3^ cells per well, whereas BALB/c-3T3 at a density of 3 × 10^3^ cells per well. HaCaT cells were incubated with increasing concentration (from 0.5 to 100 μg/mL) of both ethanol extract and FAs for 24 and 48 h, whereas BALB/c-3T3 only with ethanol extract. At the end of the incubation, cell viability was assessed by the MTT assay, as previously reported (Liberti et al. 2023). Cell viability was expressed as the percentage of viable cells in the presence of the extract compared to the controls, represented by untreated cells and cells supplemented with identical volumes of DMSO.

#### Sodium arsenite stress induction and biochemical analyses

HaCaT cells were pre-treated with 90 μg/mL of ethanol extract for 2 h. Then, cells were stressed with 300 μM sodium arsenite (NaAsO_2_) for 1 h as previously described (Sobeh et al. 2019). Immediately after NaAsO_2_ stress induction, intracellular ROS levels were determined by DCFDA assay, as previously reported (Imbimbo et al. 2023), whereas intracellular glutathione (GSH) levels were measured by performing 5,5′-dithiobis-(2-nitrobenzoic acid) (DTNB) assay, as reported by (Laezza et al. 2024).

#### Western blot analysis

Ninety minutes after stress induction, HaCaT cells were detached by trypsin and lysate in lysis buffer (0.1 M Tris HCl pH 7.4, 0.3 M NaCl, and 0.5% NP40), supplemented with proteases and phosphatases inhibitors. After 30-min incubation on ice, lysates were centrifuged at 14,000 *g* for 30 min at 4 °C. Supernatants were collected and protein concentration was determined by the Bradford assay. Eighty micrograms of proteins were separated by SDS-PAGE and analyzed by Western blotting using specific antibodies: anti-phospho-p38, anti-phospho-MAPKAPK-2 from Cell Signaling (Danvers, MA, USA); anti-Nrf2 from Bioss Antibodies (Woburn, MA, USA); anti-HO-1 from Bethyl laboratories INC. (Montgomery, TX, USA), anti-B-23, and anti-β-actin. Band detection and densitometric analyses were performed using a ChemiDoc (Biorad, Hercules, CA, USA), according to the manufacturer’s instruction.

### Statistical analyses

Samples were tested in three independent analyses, each carried out in triplicate. Results are presented as the mean of results obtained after three independent experiments (mean ± SD) and compared by one-way ANOVA according to Bonferroni’s method (post hoc) using Graphpad Prism for Windows, version 6.01.

## Results

### Pigments extraction and characterization

The proposed biorefinery strategy consists in the extraction of two different classes of molecules, pigments and lipids, starting from *P. simplex* biomass. The order of the extraction was chosen by following two different criteria: the polarity of the target molecules, to ensure that the extraction solvent would not affect the quality of the residual biomass, and the market value of the molecules to be extracted.

To obtain pigments, the biomass was harvested, freeze-dried, and treated as described in the “Materials and methods” section. At the end of the extraction, the mixture was centrifuged, the supernatant dried under N_2_ stream, and cells debris were dried and stored for further extraction. The yield of the ethanol extract was 26 ± 3%. To investigate the composition of the ethanol extract, a profiling analysis using a chromatographic HPLC–DAD system hyphenated to a QTOF-MS analyzer was used to obtain complementary structural information. UV–vis profiles and HRMS/MS data acquired in positive ionization mode (APCI +) were jointly analyzed to increase structural elucidation capacity.

**Fig. 1:**
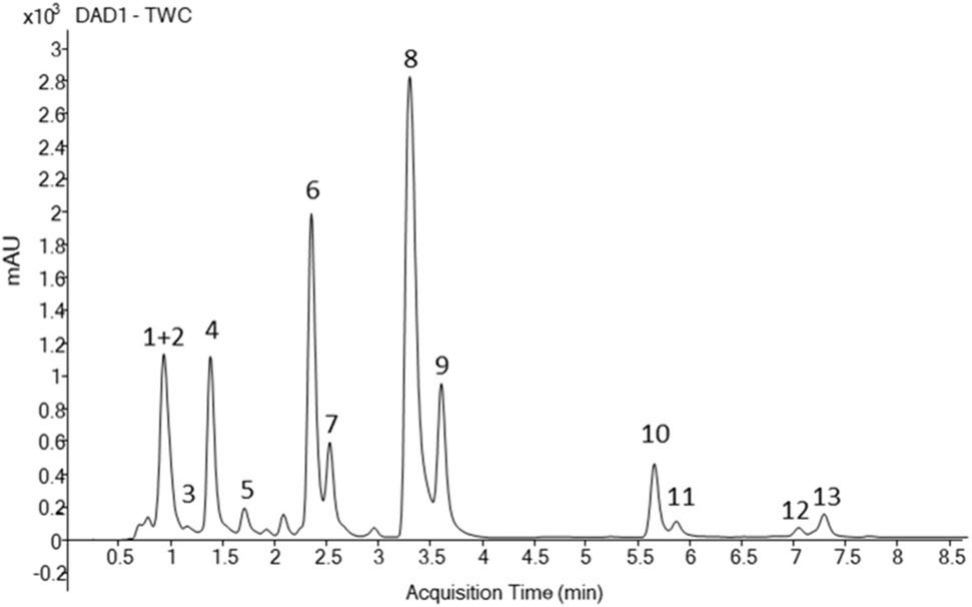
Representative LC-DAD chromatogram of pigments extracted from *P. simplex*. The TWC (total wavelength chromatogram), in the range 190 to 640 nm, is reported. Peaks identification is reported in Table S1

The UHPLC-DAD chromatographic profile, shown in Fig. 1, revealed the presence of 7 major carotenoids and 6 chlorophylls/chlorophyll derivatives, tentatively identified according to their maximum absorption wavelength (*λ*_max_), molecular ion (*m/z*), and main MS/MS fragments obtained by LC-APCI( +)-MS/MS analysis. Structural information of the annotated pigments is summarized in Table S1. From their calculated molecular formulae, chromatographic peaks 1 to 5 were annotated as compounds belonging to the xanthophylls (oxygen-containing carotenoids), whereas compounds 12 and 13 were classified as carotenes (hydrocarbon carotenoids). The nitrogen-containing pigments (peaks 6 to 11) were classified as chlorophylls and chlorophyll derivatives. Concentration values were estimated for the identified carotenoids following a semi-quantitative approach using β-carotene as reference standard (Table 1).

**Table 1:** Concentration values (mg/g_extract_) of carotenoids identified in the ethanol extract of *P. simplex*

Peak N°	Compound	Concentration (mg/g_extract_)
1 + 2	Diatoxanthin/monadoxanthin/neoxanthin	30 ± 1
3	Mutatoxanthin-type	2.5 ± 0.2
4	Lutein	30 ± 1
5	Crocoxanthin	6 ± 1
12	α-Carotene	1.6 ± 0.3
13	β-Carotene	4.6 ± 0.1

Carotenoids 1–5, 12, and 13 showed three typical maximum absorption wavelengths ranging from 400 to 475 nm in their UV–vis spectra. Compounds 1 and 2 are two major carotenoids in *P. simplex*, that coelute under the same peak and show molecular ions at *m/z* 567.4196 (C_40_H_54_O_2_) and *m/z* 601.4251 (C_40_H_56_O_4_), respectively. Compound 3 with *m/z* 585.4302 (C_40_H_56_O_3_) shares similar UV–vis absorption profile to compound 2. The first compound was annotated as a didehydro-carotenediol (e.g., diatoxanthin/monadoxanthin), whereas the second and the third were annotated as neoxanthin and the third one as mutatoxanthin-type, respectively; two biosynthetically related epoxycarotenoids and β-carotene derivatives. Peak 4 (*m/z* 569.4353, C_40_H_56_O_2_) was unambiguously annotated as lutein, the most abundant carotenoid identified in the ethanol extract of *P. simplex*, whereas peak 5 (*m/z* 551.4247, C_40_H_54_O) was tentatively identified as crocoxanthin. Both compounds 4 and 5 are biosynthetic derivatives of α-carotene. Additionally, two non-oxygenated carotenoid isomers (peaks 12 and 13) were identified as α- and β-carotene (*m/z* 537.4455, C_40_H_56_) and exhibited a higher retention time, in agreement with their higher lipophilicity. Chlorophylls and their derivatives exhibit two major absorption bands in the visible range, corresponding to the cyclic tetrapyrrole (porphyrin) skeleton, at around 420–460 nm and above 650 nm. Due to operational restrictions, only the first band could be measured in this work. In agreement with *λ*_max_ values in literature (Almela et al. 2000), compounds 6–7 and 8–9 were annotated as chlorophyll *b* and chlorophyll *a* isomers, corresponding to molecular ions at *m/z* 907.5218 (C_55_H_70_MgN_4_O_6_) and *m/z* 893.5426 (C_55_H_72_MgN_4_O_5_), respectively. Two additional chlorophyll derivatives, lacking the central Mg-atom, were annotated as pheophytin *a* isomers (compounds 10–11). These demetallized forms are less polar than the corresponding chlorophylls, showing higher retention time in reverse phase columns. The most abundant fragment ions in MS/MS spectra of chlorophyll and its derivatives usually correspond to the fragmentation with the loss of the phytyl chain [M-278]^+^.

### Lipid extraction and characterization

**Fig. 2:**
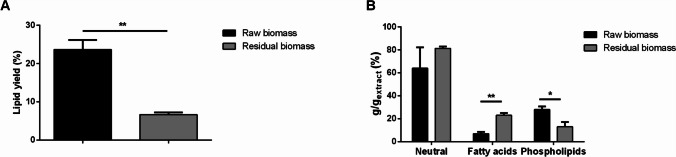
Lipid extraction and fractionation. **a** Yields of lipids extracted from *P. simplex* and reported as % with respect to dry weight biomass; **b** Yield of neutral lipids, fatty acids and phospholipids obtained upon SPE and reported as % with respect of dry weight extract. Black bars refer to raw biomass; gray bars refer to the residual biomass after pigments extraction. Results are reported as means ± SD of at least three independent experiments. * Indicates *p* < 0.05; ** indicates *p* < 0.001. The lines above the bars indicate the samples compared for statistical analysis

Lipids were extracted as the second class of molecules by an organic solvent extraction. The extraction was carried out on the residual biomass (i.e., biomass recovered after pigment extraction) after a drying step. In a parallel experiment, lipids were extracted also from the raw biomass, as benchmark, to verify if the lipids extraction could be affected by the previous pigment extraction. As shown in Fig. 2a, the yield of the hydrophobic fraction obtained from the residual biomass was 7 ± 1% (gray bar). This value represents a threefold decrease compared to the extraction yield of the raw biomass (black bar, 24 ± 3%), thus suggesting that lipids extraction was significantly affected from the pigment extraction.

To understand the composition of the isolated lipids, a solid-phase extraction (SPE) was carried out to isolate the three lipid classes: neutral lipids, fatty acids, and phospholipids. As shown in Fig. 2b, no significant alteration in neutral lipids was observed, whereas phospholipids significantly decreased (from 28 to 13%). However, it is interesting to notice a significant increase in relative fatty acid content in the extract obtained from the residual biomass (24 ± 1%) in comparison with the one obtained from the raw one (7 ± 2%).

Finally, a gas chromatography analysis was performed on fatty acids fraction obtained from raw and residual biomass. The results, reported in Table 2, suggest that *P. simplex* biomass is enriched in saturated fatty acids (SFA), particularly palmitic and stearic acids. Notably, when fatty acids were recovered from the residual biomass, the total SFA content increased, to such an extent that the extract appears to be composed only of palmitic and stearic acids.

**Table 2:** Fatty acids composition by gas chromatography analysis on samples obtained from raw and residual biomass after pigment extraction. Saturated fatty acids (SFA), monounsaturated fatty acids (MUFA), and polyunsaturated fatty acids (PUFA) are reported as relative percentages

Fatty acids		Raw biomass (%)	Residual biomass (%)
SFA			
14:0	Myristic	0.56 ± 0.08	0.4 ± 0.1
16:0	Palmitic	30.0 ± 0.8	62 ± 2
18:0	Stearic	15 ± 1	37 ± 2
24:0	Lignoceric	0.13 ± 0.04	0.02 ± 0.01
MUFA			
16:1n7t	*Trans* palmitoleic	0.04 ± 0.01	0.09 ± 0.01
16:1n7	Palmitoleic	0.20 ± 0.02	0.05 ± 0.04
18:1n9t	*Trans* oleic	0.13 ± 0.05	0.04 ± 0.01
18:1n9	Oleic	12 ± 1	0.30 ± 0.08
20:ln9	Eicosenoic	0.09 ± 0.01	0.11 ± 0.03
24:ln9	Nervonic	0.03 ± 0.03	0.010 ± 0.001
PUFA			
18:2n6t	*Trans* linoleic	0.21 ± 0.08	0.06 ± 0.04
18:2n6	Linoleic	20 ± 1	0.24 ± 0.06
18:3n6	γ-Linoleic	21.0 ± 0.8	0.10 ± 0.03
18:3n3	α-Linoleic	0.06 ± 0.01	0.07 ± 0.04
20:2n6	Eicosadienoic	0.73 ± 0.17	0.03 ± 0.03
20:3n6	Dihomo γ-Linoleic	0.03 ± 0.01	0.20 ± 0.02
20:4n6	Arachidonic (AA)	0.06 ± 0.03	0.02 ± 0.01
20:5n3	Eicosapentaenoic (EPA)	0.02 ± 0.01	0.04 ± 0.02
22:4n6	Docosatetraenoic	0.06 ± 0.04	0.010 ± 0.001
22:5n6	Docosapentaenoic-n6	0.05 ± 0.04	0.01 ± 0.01
22:5n3	Docosapentaenoic-n3	N.D	0.01 ± 0.01
22:6n3	Docosahexaenoic (DHA)	N.D	0.010 ± 0.001

### Biological characterization

#### In vitro anti-aging activity

Skin aging is a complex mechanism which depends on endogenous and exogenous factors, such as physiological aging and the continuous exposure to stress factors (Gu et al. 2020). Thus, there is a direct link between aging and oxidative stress. Different enzymes are involved in skin aging, such as tyrosinase and elastase. The first is involved in the early steps of melanogenesis, whereas the second is involved in elastin degradation, a protein that plays a key role in the maintenance of the elasticity of the overall skin tone (Panwar et al. 2020). The inhibition of these two enzymes can be a way to slow down skin aging, so that a possible inhibitor effect of *P. simplex* carotenoids extract was evaluated in vitro. The carotenoids extract was able to inhibit both enzymes, but with a lower extent with respect to kojic acid, a commercial inhibitor. The amount of extract needed to inhibit 50% of the enzyme activity (IC_50_) was calculated and reported in Table 3.

**Table 3:** Anti-tyrosinase and anti-elastase activity of *P. simplex* carotenoids extract. Values are reported as IC_50_ (mg/mL). Data shown are means ± S.D. of three independent experiments

Assay	Kojic acid IC_50_ (mg/mL)	*P. simplex* extract IC_50_ (mg/mL)
Anti-tyrosinase	0.072 ± 0.003	0.48 ± 0.05
Anti-elastase	0.116 ± 0.005	0.55 ± 0.04

#### Effect of carotenoid extract on cell viability

**Fig. 3:**
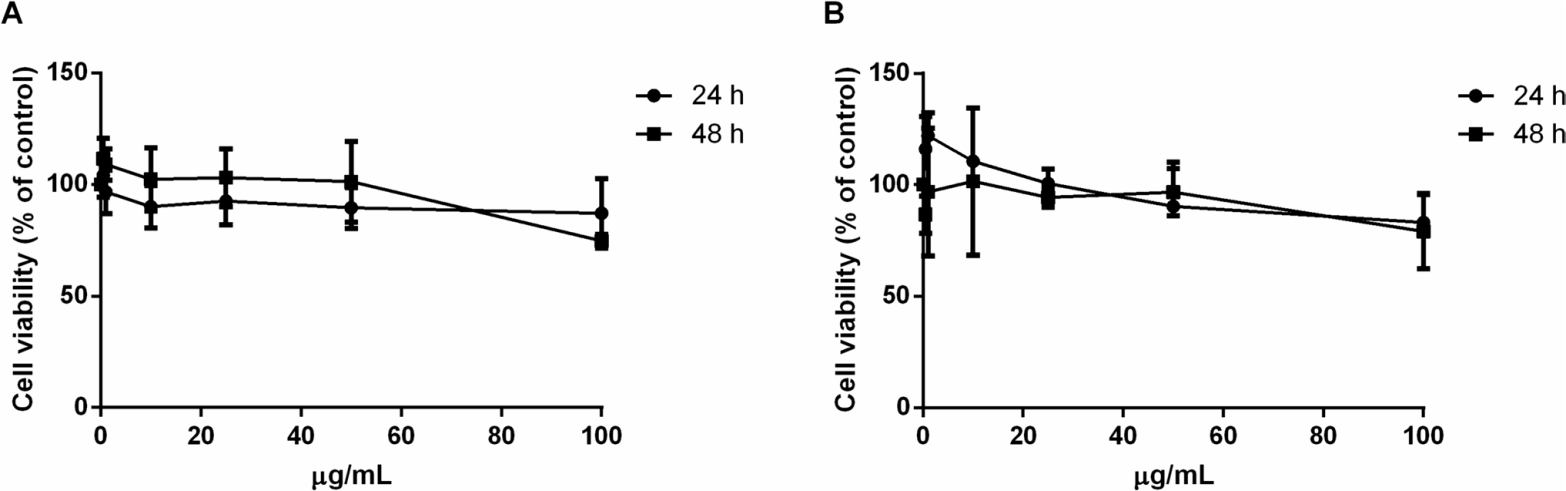
Cell viability of carotenoid extract on eukaryotic cells. HaCaT cells (**a**) and BALB/c-3T3 (**b**) incubated for 24 h (black circles) or 48 h (black squares) with increasing concentrations (0.5–100 μg/mL) of the extract. Cell viability is expressed as a percentage of viable cells in the presence of carotenoids with respect to control cells grown in the absence of the extract. Data shown are means ± S.D. of three independent experiments

The biocompatibility of the carotenoid extract was evaluated on immortalized human keratinocytes (HaCaT) and on immortalized murine fibroblasts (BALB/c-3T3) by dose- and time-dependent tests. Cell viability was assessed by the tetrazolium salt colorimetric (MTT) assay, and cell viability was expressed as the percentage of viable cells in the presence of the extract compared to that of control samples. As shown in Fig. 3, the extract was fully biocompatible on both HaCaT (Fig. 3a) and BALB/c-3T3 cells (Fig. 3b), as no reduction in cell viability was observed at any of the experimental conditions tested.

#### Protective effect of carotenoid extract against sodium arsenite-induced oxidative stress

**Fig. 4:**
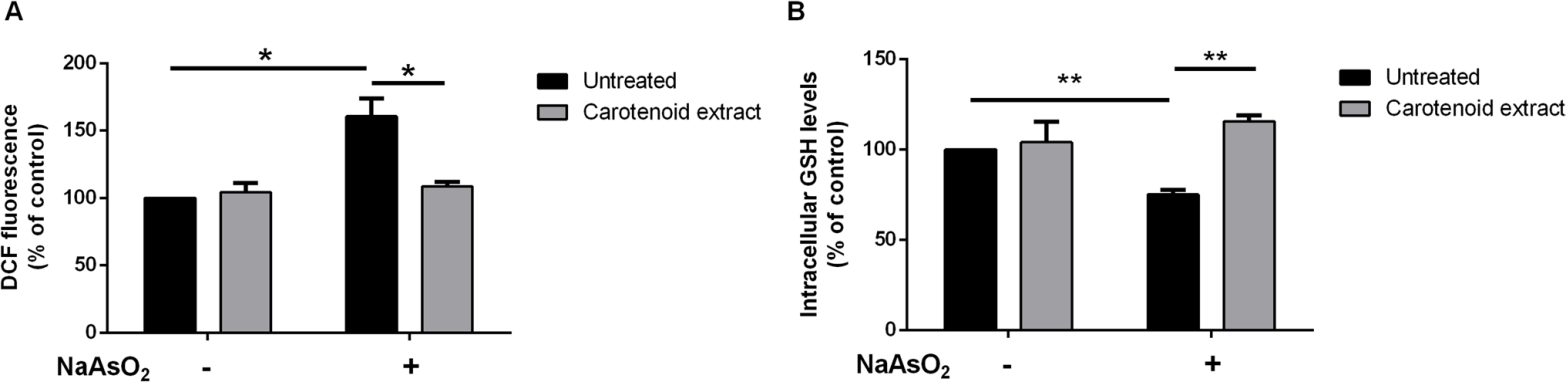
Effect of carotenoid extract on stressed HaCaT cells. Cells were incubated with 90 μg/mL of *P. simplex* carotenoid extract for 2 h in the absence ( −) or in the presence ( +) of oxidative stress induced by incubating cells with 300 μM NaAsO_2_ for 1 h. **a** Intracellular ROS levels measured by DCFDA assay; **b** intracellular GSH levels measured by DTNB assay. Values are expressed as fold increase with respect to untreated cells. Data shown are means ± S.D. of three independent experiments. * Indicates *p* < 0.05; ** indicates *p* < 0.001. Lines above the bars indicate the samples compared for statistical analysis

It is known that carotenoids are excellent antioxidants. Thus, their protective effect was tested on a cell-based system, in which cells were stressed with sodium arsenite. Humans are constantly exposed to this contaminant *via* ingestion, inhalation, and also skin absorption (Ozturk et al. 2022), with severe health problems, such as cancer, cardiovascular disease, diabetes, and skin diseases (Rahaman et al. 2021). All these conditions are triggered by oxidative stress (Sharifi-Rad et al. 2020). Thus, HaCaT cells were treated as described in the “Materials and methods” section, and immediately after NaAsO_2_-stress induction, intracellular ROS levels were measured by using the fluorescent probe 2’,7’-dichlorofluorescin diacetate (H_2_-DCFDA). As shown in Fig. 4a, in the absence of oxidative stress, no alteration in ROS production was observed when cells were treated with the carotenoid extract, whereas a significant increase in intracellular ROS levels was observed when cells were stressed with NaAsO_2_. Interestingly, when cells were pre-incubated with ethanol extract prior to stress exposure, an inhibition in ROS production was observed. The protective effect against oxidative stress was confirmed by analyzing the intracellular glutathione levels, a molecule which is normally oxidized during oxidative stress. The intracellular GSH levels were assessed using the 5,5’-dithiobis-2-nitrobenzoic acid (DTNB) assay. As shown in Fig. 4b, the exposure of the cells to NaAsO_2_ resulted in a significant GSH depletion. Nevertheless, the pre-treatment with carotenoids resulted in the inhibition of GSH depletion, thus confirming the protective effect of the extract against NaAsO_2_-induced oxidative stress.

#### Activation of Mitogen-Activated Protein Kinases (MAPK) Mediated by Carotenoid Extract

**Fig. 5:**
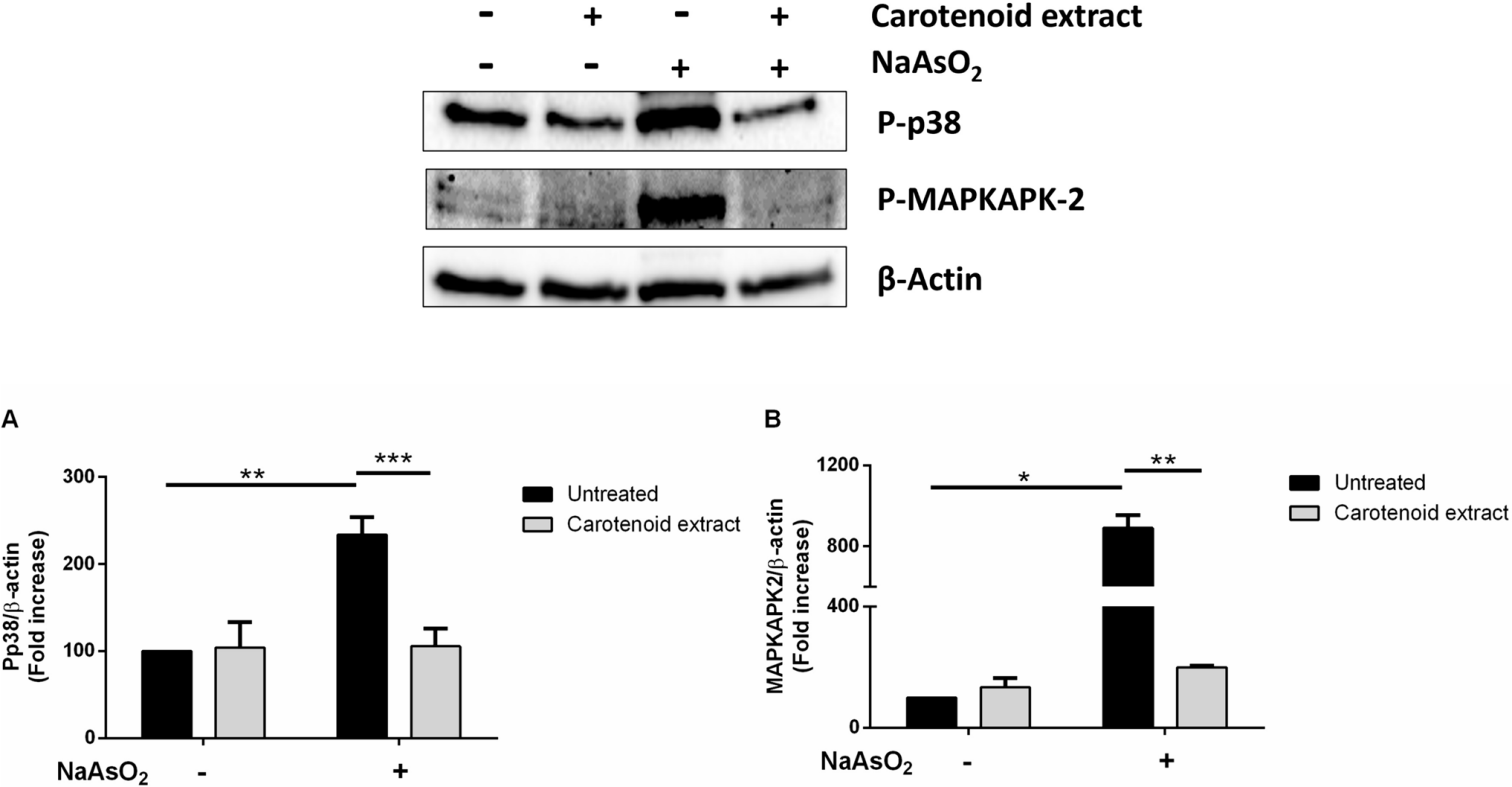
Effect of carotenoid extract on the activation of mitogen-activated protein kinase (MAPK) cascade upon NaAsO_2_ stress induction. HaCaT cells were treated as described in the “Materials and methods” section. Phosphorylation levels of p38 and MAPKAPK-2 were analyzed by Western blotting. β-Actin was used as an internal standard. The relative densitometric analysis of P-p38 (**a**) and P-MAPKAPK-2 (**b**) levels is reported. Black bars refer to control cells in the absence (–) or in the presence ( +) of sodium arsenite; light gray bars refer to cells incubated with 90 μg/mL of carotenoids. Data shown are the means ± S.D. of three independent experiments. * Indicates *p* < 0.05, ** indicates *p* < 0.01, *** indicates *p* < 0.001. Lines above the bars indicate the samples compared for statistical analysis

Oxidative stress usually results in the activation of MAP (mitogen-activated protein) kinases pathway. Following oxidative stress insult, p38 is phosphorylated and this causes, in turn, the phosphorylation of its direct target, MAPKAPK-2. Thus, Western blotting analyses were performed to evaluate the protective effect of carotenoids extracted from *P. simplex* biomass. As shown in Fig. 5, the carotenoid extract was able to inhibit the phosphorylation of both p38 and MAPKAPK-2. A complete inhibition in the phosphorylation of p38 and MAPKAPK-2 levels was observed when cells were pre-treated with the extract prior to be stressed.

#### Protection against oxidative stress *via* the activation of Nrf2 pathway

**Fig. 6:**
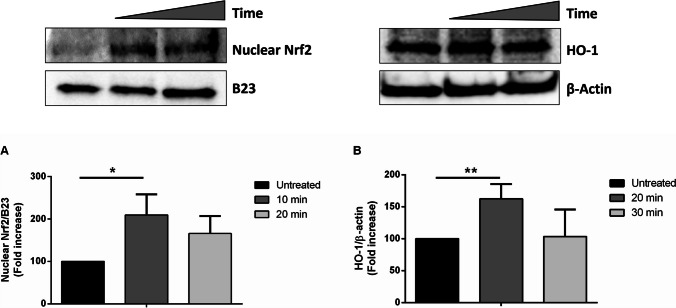
Effect of carotenoid extract on Nrf2 activation. HaCaT cells were incubated with 90 μg/mL of ethanol extract for different length of times. **a** Western blot analysis of nuclear Nrf2 of untreated cells (black bars), after 10 min (dark gray bar) and 20 min (light gray bar) incubation. **b** Western blot analysis of cytosolic HO-1 of untreated cells (black bars), after 20 min (dark gray bar) and 30 min (light gray bar) incubation. Anti-B23 antibody was used as internal standard for nuclear lysate, whereas anti-β-actin antibody was used for cytosol. Images were quantified by densitometric analysis. Data shown are means ± S.D. of three independent experiments. * Indicates *p* < 0.05, ** indicates *p *< 0.01  with respect to control cells. Lines above the bars indicate the samples compared for statistical analysis

The nuclear factor E2-related factor 2 (Nrf2) plays a pivotal role in cellular responses to oxidative stress (Li and Kong 2009), as it regulates the expression of antioxidant enzymes and maintains the redox homeostasis (Mansouri et al. 2022). Under physiological conditions, Nrf2 is in the cytosol, associated with the kelch-like ECH-associated protein 1 (KEAP1). In the presence of external stimuli, such as oxidative stress or low exposure to antioxidants, Nrf2 dissociates from KEAP1 and rapidly translocates into the nucleus where it binds the antioxidant responsive elements (ARE) sequences, thus initiating the transcription of over 200 genes involved in the antioxidant response, among which heme oxygenase 1 (HO-1) (Mallard et al. 2020). For this reason, the effect of carotenoid extract in the activation of Nrf2 pathway was analyzed. HaCaT cells were treated with 90 μg/mL of carotenoid extract for 10 or 20 min, and then, lysates were analyzed by Western blot analysis, using Nrf2 antibody. As shown in Fig. 6a, a significant increase in nuclear Nrf2 levels was observed upon 10-min incubation. To corroborate this result, HO-1 levels were also analyzed by Western blot, upon 20- and 30-min incubation. As shown in Fig. 6b, a significant increase in HO-1 levels was observed after 20 min incubation.

#### Biocompatibility of FAs

**Fig. 7:**
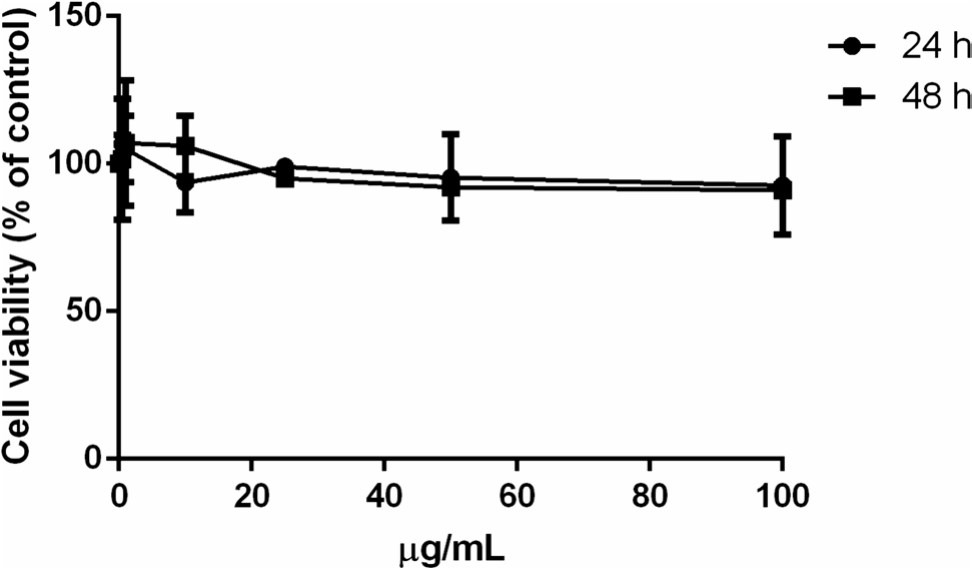
Biocompatibility of isolated FAs on human keratinocytes. HaCaT cells were incubated for 24 h (black circles) or 48 h (black squares) with increasing concentrations (0.5–100 μg/mL) of FAs isolated from the residual biomass of *P. simplex*. Cell viability is expressed as a percentage of viable cells in the presence of the extracts under test with respect to control cells grown in the absence of the FAs. Data shown are means ± S.D. of three independent experiments

Finally, the biocompatibility of palmitic and stearic acids was tested on HaCaT cells. In particular, the FAs obtained at the end of the biorefinery were analyzed following the same procedure used above. As shown in Fig. 7, no effect on cell viability was observed at any of the experimental conditions under test, thus indicating the safeness of the isolated FAs.

## Discussion

In the last years, the use of natural molecules to be used in the cosmeceutical field is emerging and the biotech industries are focusing their attention on safe, sustainable, and economical natural sources. The blue biotechnology, based on the use of aquatic resources as raw materials, is meeting consumer demands for natural active molecules to be used in cosmetics and cosmeceuticals. In this context, microalgae represent a versatile reservoir of active molecules with many potential applications, ranging from antioxidants (Imbimbo et al. 2019), anticancer (Ferraro et al. 2020) to anti-aging (Khemiri et al. 2023), as its biomass contains an array of valuable metabolites, such as proteins, lipids, polysaccharides, pigments and vitamins, all known for their antioxidant, anti-aging, and moisturizing activities.

However, several drawbacks hinder microalgae large-scale use, which eventually lead to an increase in overall costs (Imbimbo et al. 2020; Abdur Razzak et al. 2023). A possible solution to lower the costs is the biorefinery approach: a stepwise extraction of more than one product to reduce the environmental footprint (Chanana et al. 2023). This should be combined to eco-friendly extraction techniques to obtain biologically active and safe extracts, in terms of negligible cytotoxicity.

Here, an unexplored green microalga strain has been used to isolate different classes of molecules with biological activity. Starting from *Pseudococcomyxa simplex* biomass, carotenoids were extracted first using a green approach. It is well known that carotenoids can exert a potent antioxidant activity, thus protecting skin cells from stress agents and improving skin appearance (Mussagy et al. 2023). Lutein, the most abundant one, is a high added value carotenoid with reported anticancer, antibacterial, anti-neurodegenerative, and anti-inflammatory activities (Desai and Mane 2024), sold at 455,000 €/g. The obtained extract shows a strong antioxidant activity and can activate the Nrf2 pathway, which has been primarily identified as a key player in the antioxidant response. The activation of Nrf2 pathway is also involved in the modulation of skin pigmentation, in wound healing, and in the overall protection of the skin from the environmental stresses, so that the use of Nrf2 modulators is considered a useful tool in dermo-cosmetic applications. Recent studies linked the pivotal role of Nrf2 to other key pathways, such as anti-inflammatory and skin aging (Frantz et al. 2023). Wrinkles, loss of elasticity, hyperpigmentation, and dryness are classical symptoms of skin aging, mainly due to the activity of key enzymes, such as those involved in elastin degradation or in the early steps of melanogenesis (Shin et al. 2023). In particular, melanin overproduction can cause different dermatological problems, such as hyperpigmentation, which can finally lead to melanoma (Bayrakçeken Güven et al. 2023). Our data strongly support the connection between antioxidants, Nrf2 activation, and skin aging, as the extract shows an inhibitory effect towards tyrosinase and elastase enzymes. Our results are in line with those reported in literature on carotenoids extracted from different strains, such as astaxanthin (Mourelle et al. 2017; Dutta et al. 2023), lutein (Jiang et al. 2024), and β-carotene (Yeager and Lim 2019). Accordingly, microalgae extracts are currently being formulated into skin-care products for providing anti-aging, moisturizing, antioxidant, and anti-irritant benefits (Desai and Mane 2024). The lipids isolated from the residual biomass belong to saturated fatty acids, a neglected class of lipids which are now more considered, as they can find application as antibacterial molecules, in cosmetics and in drug delivery (Liberti et al. 2022). Lipid yield obtained from the raw biomass (24%) is in agreement with the literature (Santhakumaran et al. 2018), whereas lipid yield from the residual biomass is almost three times lower, probably because of the first extraction with ethanol. However, from the residual biomass, a pure class of fatty acids is obtained by a single purification step. We can hypothesize that lipid yield is affected by the first extraction solvent: the more hydrophobic solvent is used for the first extraction, the lower is the lipid yield obtained from the residual biomass. According to this hypothesis, when an aqueous buffer is used as the first extraction solvent, an increase in the lipid yield is observed during the further extraction, as all the lipophilic molecules cannot be extracted by the polar solvent. Moreover, if lipid recovery is preceded by an ethanol extraction, the lipid content decreases, as some class of lipids (those with a lower hydrophobicity) can be co-extracted by ethanol (Imbimbo et al. 2019; Liberti et al. 2022), and an increase in SFA content is observed (Liberti et al. 2022). In cosmetics, fatty acids can be used as oily raw materials, as emulsifiers, and as softeners because they can deposit between desquamating cells (De Luca et al. 2021). The isolated fatty acids were fully biocompatible, thus suggesting a possible role in cosmetics.

The main objective of cosmetic formulations is to use extracts endowed with antioxidant, anti-aging, and moisturizing potential, so that microalgae fulfill these requirements. They also represent a sustainable vegan alternative to replace potentially harmful chemicals, traditionally used in skin care products. Algae molecules can protect from aging, UV, and oxidative stress, but only few strains are commercialized. Currently, molecules or extracts from *Chlorella vulgaris*, *Haematococcus pluvialis*, *Dunaliella salina*, *Nannochloropsis oculata*, and *Phaeodactylum tricornutum* are normally employed, so that the exploration of untapped species is now mandatory. This study represents a milestone but also a starting point for process improvement. Indeed, biomass concentration, advances in extraction technologies, formulation strategies, and a detailed cost analysis are expected to accelerate the adoption of *P. simplex* in the cosmetics industry.

## Supplementary Information

Below is the link to the electronic supplementary material.

Supplementary file1 (PDF 539 KB)

## Data Availability

All data generated during this study are included in this published article and its supplementary information file.
