# Supplementary material for: Unveiling the potential of Pseudococcomyxa simplex: a stepwise extraction for cosmetic applications
Source: Appl Microbiol Biotechnol. 2024 Jun 24;108:390. doi: 10.1007/s00253-024-13229-9 (PMC11194203; doi:10.1007/s00253-024-13229-9)
Supplement: Supplementary file 1 [file 253_2024_13229_MOESM1_ESM.pdf]

Supplementary Information

Unveiling the potential of *Pseudococcomyxa simplex*: a stepwise extraction for cosmetic applications

Paola Imbimbo[1*], Enrica Giustino[1], Alfonso Ferrara[1], Gerardo Alvarez-Rivera[2], Hassan Annaz[3], Elena Ibanez[2], Maria Chiara Di Meo[4], Armando Zarrelli[1], Daria Maria Monti[1*]

[1] Department of Chemical Sciences, University of Naples Federico II, via Cinthia 4, 80126, Naples, Italy.

[2] Laboratory of Foodomics, Institute of Food Science Research, CIAL, CSIC, Nicolás Cabrera 9, 28049 Madrid, Spain.

[3] AgroBioSciences Program, College of Agriculture and Environmental Science, University Mohammed VI Polytechnic, Ben Guerir, Morocco.

[4] Department of Sciences and Technologies (DST), University of Sannio, BN, Benevento, 82100, Italy.

*Corresponding author: Paola Imbimbo, paola.imbimbo@unina.it; Daria Maria Monti, mdmonti@unina.it

Table S1. Tentatively identified carotenoids and chlorophylls in *Pseudococcomyxa simplex* by HPLC-DAD-APCI-QTOF analysis, including peak annotation, high-resolution mass spectrometry features and UV–Vis maxima.

Peak Nº	RT (min)	Identification	Molecular formula	Monoisotopic mass	Theoretical $[M + H]^+$ m/z	Error (ppm)	MS/MS product ions	UV–Vis maxima (nm)	% of total pigments
1	0.815	Diatoxanthin/Monadoxanthin	$C_{40}H_{54}O_2$	566.4123	567.4196	2.2	549, 427, 121	424s, 447, 477	13 ± 1
2	0.922	Neoxanthin	$C_{40}H_{56}O_4$	600.4179	601.4251	2.1	583, 333, 167	417, 440, 467	
3	1.095	Mutatoxanthin-type	$C_{40}H_{56}O_3$	584.4229	585.4302	1.2	567, 476, 133	417, 440, 467	1 ± 0.1
4	1.315	Lutein*	$C_{40}H_{56}O_2$	568.4280	569.4353	3.0	551, 431, 337	422s, 445, 473	13 ± 1
5	1.642	Crocoxanthin	$C_{40}H_{54}O$	550.4175	551.4247	4.6	533, 495, 175	417s, 440, 467	2.6 ± 0.6
6	2.289	Chlorophyll *b*	$C_{55}H_{70}MgN_4O_6$	906.5146	907.5219	1.8	630, 601, 569	468	16.8 ± 0.1
7	2.462	Chlorophyll *b*	$C_{55}H_{70}MgN_4O_6$	906.5146	907.5219	2.4	630, 601, 569	468	5.1 ± 1.3
8	3.235	Chlorophyll *a*	$C_{55}H_{72}MgN_4O_5$	892.5353	893.5426	4.5	615, 583, 555	339, 390s, 420s, 432	37.3 ± 4.5
9	3.542	Chlorophyll *a*	$C_{55}H_{72}MgN_4O_5$	892.5353	893.5426	4.5	615, 583, 555	339, 390s, 420s, 432	6.6 ± 4.5
10	5.595	Pheophytin *a*	$C_{55}H_{74}N_4O_5$	870.5659	871.5732	2.8	593, 533	408	2.4 ± 2.6
11	5.809	Pheophytin *a*	$C_{55}H_{74}N_4O_5$	870.5659	871.5732	2.8	593, 533	408	0.6 ± 0.8
12	7.008	Alpha-carotene*	$C_{40}H_{56}$	536.4382	537.4455	3.4	457, 177, 123	425s, 446, 473	0.7 ± 0.1
13	7.248	Beta-carotene*	$C_{40}H_{56}$	536.4382	537.4455	3.4	457, 321, 203	430s, 452, 478	1.9 ± 0.1

*Confirmed by commercial standard.
